# Socioeconomic inequalities in the use of caesarean section delivery in Ghana: a cross-sectional study using nationally representative data

**DOI:** 10.1186/s12939-019-1063-6

**Published:** 2019-10-25

**Authors:** Emmanuel Dankwah, Shelley Kirychuk, Wu Zeng, Cindy Feng, Marwa Farag

**Affiliations:** 1 School of Public Health, University of Saskatchewan, 104 Clinic Place, Saskatoon, SK S7N 2Z4 Canada; 2 Canadian Centre for Health and Safety in Agriculture (CCHSA), College of Medicine, University of Saskatchewan, Saskatoon, SK S7N 2Z4 Canada; 3 Department of International Health, School of Nursing & Health Studies, Georgetown University, 37th and O Streets, N.W, Washington, DC 20057 USA; 4 School of Epidemiology and Public Health, Faculty of Medicine, University of Ottawa, 600 Peter Morand, Ottawa, Ontario K1G 5Z3 Canada; 5 School of Public Administration and Development Economics, Doha Institute for Graduate Studies, Al Tarfa Street, Zone 70, Doha, Qatar

**Keywords:** Caesarean section, Birth, Delivery, Socio-economic, Inequities, Ghana

## Abstract

**Background:**

Inappropriate use of Caesarean Section (CS) delivery is partly to blame for Ghana’s high maternal mortality rate. However, previous research offered mixed findings about factors associated with CS use. The goal of this study is to examine use of CS in Ghana and the socioeconomic factors associated with it.

**Methods:**

Data from the nationally representative 2014 Ghana Demographic and Health Survey (GDHS) was used after permission from the Monitoring and Evaluation to Assess and Use Results (MEASURE) Demographic and Health Survey (DHS) program. Univariable and multivariable logistic regression models were fitted to examine the socioeconomic inequalities in CS use. The independent variables included maternal age, marital status, religion, ethnicity, education, place of residence, wealth quintile, and working status. Concentration index (CI) and rate-ratios were computed to ascertain the level of CS inequalities.

**Results:**

Out of the 4294 women, 11.4% had CS delivery. However, the percentage of CS delivery ranged from 5% of women in the poorest quintile to 27.5% of women in the richest qunitle. Significant associations were detected between CS delivery and maternal age, parity, education, and wealth quintile .

**Conclusions:**

This study revealed that first, even though Ghana has achieved an aggregate CS rate consistent with WHO recommendations, it still suffers from inequities in the use of CS. Second, both underuse of CS among poorer women in Ghana and overuse among rich and educated women are public health concerns that need to be addressed. Third, the results show in spite of Ghana’s free maternal care services policies, wealth status of women continues to be strongly and signtificantly associated with CS delivery, indicating that there are indirect health care costs and other reasons preventing poorer women from having access to CS which should be understood better and addressed with appropriate policies.

## Introduction

Caesarean Section (CS) is a life-saving obstetric surgical intervention for mothers and babies [1, 2]. This vital clinical procedure is often needed as a result of several medical conditions including macrosomia, pregnancy-induced hypertension, maternal weight, among others [3–5]. Yearly, about 18.5 million CS births are recorded worldwide, [6] this CS rate constitutes an average 19.1% of total births with great variations across the geographic regions [7–9].

This CS disparity represent underuse or possibly medically unjustified overuse [7, 10–13]. Studies have posited that unlike higher-income countries that have adequate or even overuse, C-Sections in lower-income regions of the world are persistently underutilized [11, 14, 15] though developing countries represents 60% of global births, [6]. Irani and Deering [16] reported a 3.6% CS rate in sub-Saharan Africa.

Globally, inequities in the use of CS for delivery have seriously affected maternal and newborn health outcomes [11, 14]. However, most studies have linked inadequate access to CS delivery to maternal and newborn deaths and morbidities especially in resource limited settings [11, 14, 15]. Further, Ahmed and Tuncalp [17] related poor maternal and perinatal health outcomes to delay or lack of timely caesarean intervention; which has consequences such as stillbirth, ruptured uterine, obstetric fistula and many other obstetric conditions.

Recent studies have found that out of the 3.2 million extra CS that would be required every year in low-income settings to avoid maternal and neonatal deaths, approximately 68.5% would be needed in Africa [6].

Like many developing countries, Ghana’s maternal mortality rate is high; by the end of 2015 the estimated rate was 319 maternal deaths per 100,000 live births [18]. According to 2014 mortality report, 9% of all female deaths in Ghana were pregnancy-related, [19]. Available data declared a CS rate of 6.9 in 2008 [20] with an unmet C-sections of about 23,467 per year in Ghana [6]. Though the association between CS birth and socio-economic predictors have been well-established in literature, [21–27] the inconsistent findings underpin the need for this study. Specifically, a small number of studies have been conducted in Ghana to examine CS delivery use [28–30]. However, the magnitude and direction of the socioeconomic effects on CS birth remain unclear. For example, while no significant association between CS delivery and parity was reported by Pra et al. [29]. Manye et al. [30] found that parity of the women was a strong predictor of CS delivery use. Likewise, older women (> 34 years) were more likely to use CS delivery than younger women according to Manye et al. [30] and yet the reverse is the case in the study by Prah et al. [29].

Moreover, most of the existing Ghanaian studies on CS births are hospital-based and some failed to sample participants randomly. This obviously has the potential to bias the study estimates. For instance, Danso et al. [28] used a convenience sampling technique to select 154 women who used CS delivery from two teaching hospitals in Ghana. Similarly, Prah et al. [29] reviewed medical records from a health facility and reported CS delivery rate of 26.9%. Further, the only existing population-based study on CS delivery focused on small geographical area of the country [30], that was in just two rural districts out of the 216 districts in Ghana and therefore restricts making inference about the entire population [30]. Also, none of the studies quantified the extent of inequality using concentration index and curve. This study aims to contribute to a growing international policy-relevant body of literature examining the effect of socioeconomic factors on CS delivery [31–33] . The goal of this study is to investigate the association between CS delivery and socioeconomic factors using nationally representative data from Ghana.

## Methods

### Study data and variables

**Fig. 1:**
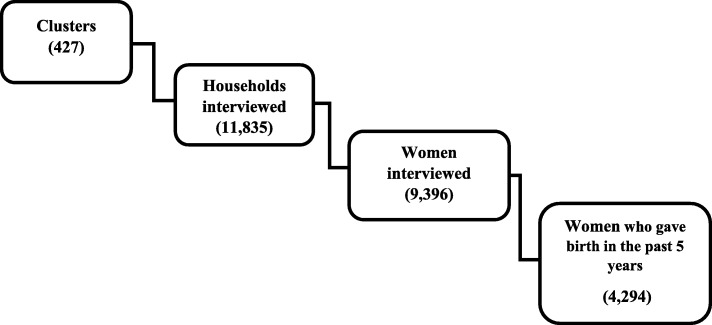
A flow chart of study participants

The data for this study were drawn from the 2014 Ghana Demographic and Health Survey (GDHS), a nationwide population-based survey. The subset of data used for this study includes only females between 15 and 49 years old residing in Ghana. Based on the updated 2010 Ghana Population and housing Census (PHC) respondents were selected in all the ten regions of the country. Participants were selected into the survey in two stages. Firstly, 427 clusters were selected and subsequently 30 households from each cluster were selected through systematic sampling. In total, 12,831 households were selected for the survey; actual interviews were conducted in 11,835 households out of the 12,010 households that were occupied representing a response rate of 98.5%. Trained interviewers used a structured questionnaire that was pretested to collect data from 9396 women on the socio-demographic background, reproductive, family planning history and other aspects of women’s health. Among eligible women, the interviewed women in GDHS recorded a 3% non-respnse rate (Fig. 1). Weighted cluster sampling was applied in the survey [34].

The 2014 GDHS gathered data on recent births in the 5 years preceding the survey. The study outcome was whether the delivery was a CS or not. Women who reported having used CS delivery were classified as “1 = CS delivery” otherwise, they were classified as “0 = not CS delivery”**.** Based on an extensive literature review, the following self-reported variables from the survey were selected and studied to assess the inequities in use of CS delivery: maternal age, marital status, religion, parity, place of residence, education, financial status, and working status [24, 25, 30].

For the analysis conducted in this study, maternal age was categorized into three groups [15–24, 25–34, 35–49]. Regarding marital status, it was grouped into single, married, cohabitating and widowed or separated or divorced. For the religious affiliation of the women, it was classified into three groups (Christians, Islam and Traditional/other). The cateogries used for the variable Ethnic group of the woman were Northern tribes, Akan, Ewe, Ga and other. The parity of the women which is the birth order was categorized into three cohorts (One birth, 2 births and ≥ 3 births). With respect to education, women were categorized into four groups: no education, primary, secondary, and higher.

For the purposes of this study, mothers’ place of residence was grouped into rural and urban. The wealth quintile of the women was ascertained using principle component analysis of assets and household factors and hence classified into poorest, poorer, middle, richer, and richest as present in other studies [35]. Finally, the working status of the women was classified into employed and unemployed.

### Statistical analysis

Frequencies and percentages were calculated for categorical variables included in this study using the Pearson Chi-square test. To examine the determinants of CS delivery, a regression model with logit link function was fitted. Univariable and multivariable analyses were conducted to estimate odds ratio (OR) and 95% confidence interval. A lenient *p*-value of 0.25 was adopted to select potential predictors into the adjusted model, mainly to avoid missing important factors in the multivariable analysis whose effect could be masked, suppressed or inflated by other controlling variables [36, 37]. Multicollinearity was tested for risk factors selected for the multivariable model, variance inflation factor (VIF) greater than 2.5 or tolerance of less than 0.4 were deemed to be collinear. A manual backward elimination selection method was used to estimate adjusted odds ratio (AOR) for CS births. In the adjusted model, explanatory variables were considered significant at *p*-value ≤0.05. Confounding and interaction effects of predictors in the adjusted model were checked. In this study, explanatory variables were included in the multivariate regression analysis if the percentage change in regression coefficient between the crude and the adjusted model was greater than 20%. Also, two-way interactions were examined between significant explanatory variables in the adjusted model. Besides, sample weighting adjustment was applied for the unequal proportion of samples from the population and differential nonresponse rate. Also, the model incorporated robust variance estimation method to ensure accurate point and interval were computed after adjusting for design characteristics. In addition, area under the curve (AUC) of the receiver operating characteristics (ROC) was computed as a postestimation goodness-of-fit test, and parsimonious model was selected using Akaike’s Information Criterion (AIC). Smaller AIC was adjudged a better model [38].

To estimate the inequality in CS birth based on wealth quintile, the following health equity metrics were used: rate-ratios, concentration curves and concentration indices [39–42]. To compute rate-ratio, the percentage CS delivery among the richest group was divided by the proportion of CS delivery among the poorest group. Furthermore, the degree of inequality was displayed graphically using the concentration curve; this was done by plotting the cumulative proportion of the population ranked by the wealth quintile of the women on the x-axis against the cumulative proportion of CS delivery on the y-axis. The diagonal line on the graph is the equality line of caesarean delivery based on wealth quintile. A concentration curve that is above the equality line indicates that CS births are concentrated among economically disadvantaged groups whereas a curve below the equality line suggests more CS delivery among the wealthier groups. The Concentration index was used to quantify the level of inequities in the use of CS delivery. The scale of concentration index ranges from − 1 to + 1, zero signifies equality in CS delivery whereas negative and positive values represent greater concentration of CS births among the lower-class and affluent groups respectively [39–41]. For clarity in the interpretation of concentration of index, Koolman and Doorslaer redistribution scheme [43] was adopted in this study to estimate the percentage redistribution of CS delivery use required to achieve a concentration index equal to zero. All the data analyses were conducted in STATA version 14.0 (Stata Corp., College Station, Texas: StataCorp LP, USA) and Microsoft excel version 16. The equation for concentration index was adopted from some studies [40, 41]:

$$ \mathrm{Concentration}\ \mathrm{index}=\left({\mathrm{p}}_1{\mathrm{L}}_2-{\mathrm{p}}_2{\mathrm{L}}_1\right)+\left({\mathrm{p}}_2{\mathrm{L}}_3-{\mathrm{p}}_3{\mathrm{L}}_2\right)+\dots +\left({\mathrm{p}}_{\mathrm{T}-1}{\mathrm{L}}_{\mathrm{T}}-{\mathrm{p}}_{\mathrm{T}}{\mathrm{L}}_{\mathrm{T}-1}\right) $$

- p_T_: the cumulative percent of the sample ranked by the socioeconomic groups
- L_T_: the corresponding concentration curve ordinate; and
- T: the number of wealth quintile groups

## Results

### Descriptive results

Out of the 4294 women that were asked whether delivery was by CS or not in the GDHS, only11.4% reported using CS for delivery. The distribution of women across predictors and mode of delivery is detailed in Table 1.

**Table 1:** Distribution of women by predictors and caesarean section (CS) delivery, and crude odds ratio and 95% confidence interval of having CS delivery in univariable model

Predictors	N (%)	CS delivery (%)	Crude OR (95% CI)	*P*-value
	4294 (100%)			
Maternal age				< 0.0001
15–24 years	923 (21.5)	6.6	ref.	
25–34 years	2026 (47.2)	11.5	2.42 (1.65, 3.55)	
35–49 years	1345 (31.3)	14.3	3.31 (2.39, 4.59)	
Marital status				0.01
Single	363 (8.5)	12.1	ref.	
Married	2801 (65.2)	11.9	1.52 (1.01, 2.28)	
Cohabitating	830 (19.3)	8.9	0.99 (0.62,1.60)	
Widow/Separated/Divorced	300 (7.0)	12.3	1.74 (0.84, 3.63)	
Religion				0.06
Traditional/other	324 (7.6)	5.6	ref.	
Islam	885 (20.6)	9.9	1.56 (0.69, 3.49)	
Christian	3085 (71.8)	12.4	1.97 (0.98, 3.96)	
Ethnicity*				< 0.0001
Northern tribes	1796 (41.8)	7.5	ref.	
Akan	1643 (38.3)	15.2	2.19 (1.62, 2.96)	
Ewe	476 (11.1)	11.3	1.50 (0.99, 2.27)	
Ga	198 (4.6)	16.2	2.93 (1.68, 5.14)	
Other	180 (4.2)	10.0	1.65 (0.85, 3.21)	
Parity				< 0.0001
1birth	935 (21.8)	16.6	ref.	
2 births	839 (19.5)	12.2	0.68 (0.51, 0.91)	
≥ 3 births	2520 (58.7)	9.2	0.54 (0.41, 0.71)	
Education				< 0.0001
No education	1419 (33.0)	6.1	ref.	
Primary	869 (20.2)	8.5	1.88 (1.15, 3.07)	
Secondary	1837 (42.8)	14.8	3.10 (2.25, 4.27)	
Higher	169 (4.0)	33.1	8.28 (5.11, 13.41)	
Place of residence				< 0.0001
Rural	2516 (58.6)	7.3	ref.	
Urban	1778 (41.4)	17.1	2.55 (1.89, 3.43)	
Wealth quintile				< 0.0001
Poorest	1318 (30.6)	5.0	ref.	
Poorer	923 (21.5)	7.2	1.56 (1.01, 2.41)	
Middle	812 (18.9)	11.1	2.67 (1.82, 3.92)	
Richer	685 (16.0)	16.5	4.21 (2.81, 6.30)	
Richest	556 (13.0)	27.5	8.12 (5.52, 11.94)	
Working status				0.8
Unemployed	886 (20.6)	11.0	ref.	
Employed	3408 (79.4)	11.4	1.03 (0.75, 1.43)	

*Abbreviations: N* number of observations, *%* percent, *N = 4293 due to missing values, *CI* confidence interval, *OR* odds ratio, *ref.* reference

The ages of the mothers were between 15 and 49 years with an average age of 29.7 years. Most (47.2%) of the mothers were aged 25–34 years. CS births among women aged 25–34 years was nearly double of those aged 15–24 years (11.5% versus 6.6%). CS delivery rate was 14.3% in older women (35–49 years), compared with 6.6% among younger women (15–24 years).

Majority (65.2%) of the women were married. Women cohabitating had the lowest proportion (8.9%) of CS births whereas women who were either widowed or divorced or separated had a CS rate little above 12%.

Nearly three-quarters of women (71.8%) were Christians. CS births for Christians were about twice that of the traditional and other believers (12.4% versus 5.6%). On the other hand, the proportion of Muslim women who reported having had a CS was 9.9%.

With respect to ethnic orientation, Northern tribes women were 41.8% while Akans were 38.3%. Minority groups constituted about 20% of the women; they were Ewe (11.1%), Ga (4.6%) and other ethnic groups. CS delivery ranged from a lowest rate of 7.5% among northern tribes to 16.2% in Ga women.

Around 58.7% of the mothers had at least 3 births. Parity of the women had inverse relationship with CS delivery. CS rate decreases as birth order increases: CS delivery among mothers with one birth reduced from 16.6 to 12.2% (2 births) and 9.2% (3 or more births).

One-third (33.0%) of the women had no formal education whilst only 4.0% attained post-secondary education. For women with no education, the proportion of women who had a CS delivery was 6.1% whereas the CS prevalence among women with primary education was 8.5, 14% among women with secondary education, and 33.1% among women with higher education.

More than half (58.6%) of the women resided in the rural settings of Ghana. The results show that CS delivery rate was more than two-folds higher in urban dwellers than women living in rural communities (17.1% versus 7.3%).

The analysis revealed that an increase in the wealth status of women had a corresponding increase in CS rate. Just over one-quarter (27.5%) of the richest group had CS birth whilst the percentage was 5% for the poorest group.

Out of 5 mothers, about four were employed (79.4%). There was no substantial difference in the proportion of CS births among employed and unemployed women (11.4% versus 11.0%).

### Univariable model results

The crude association between mode of delivery and predictors are displayed in Table 1. The results revealed that except women’s working status that was excluded, all the explanatory variables were selected for further analysis in the multivariable model at a liberal *p*-value of 0.25.

### Multivariable model results

The analysis did not find significant interaction and multicollinearity among the select predictors in the multivariable model. The multivariable associations between mode of delivery and associated factors are presented in Table 2. In the adjusted model, women’s religion, ethnicity, place of residence and marital status were not statistically significant at *p*-value of 0.05 when all factors were controlled. Predictors that had a significant effect on CS delivery were maternal age, parity, education level, and wealth quintile.

**Table 2:** AORs and corresponding 95% CIs of having a caesarean delivery by predictors in the multivariable logistic regression model

Predictors	AOR (95% CI)	*P*-value
Maternal age (ref. 15–24 years)		
25–34 years	3.15 (2.11, 4.71)	< 0.0001
35–49 years	7.53 (5.11,11.08)	< 0.0001
Parity (ref. one birth)		
2 births	0.52 (0.38, 0.73)	< 0.0001
≥ 3 births	0.31 (0.22, 0.43)	< 0.0001
Education (ref.no education)		
Primary	1.59 (0.98, 2.59)	0.06
Secondary	1.65 (1.15, 2.36)	0.006
Higher	2.17 (1.26, 3.74)	0.005
Wealth quintile (ref. poorest)		
Poorer	1.36 (0.89, 2.06)	0.2
Middle	2.13 (1.43, 3.18)	< 0.0001
Richer	2.76 (1.77, 4.28)	< 0.0001
Richest	4.38 (2.83, 6.77)	< 0.0001

*Abbreviations: CI* confidence interval, *AOR* adjusted odds ratio, *%* percent, *ref.* reference

This study detected positive association between CS birth and maternal age. Mothers aged 25–34 years were 3.15 (95% CI = 2.11–4.71) times more likely to have had a CS delivery relative to women with ages 15 to 24 years. Similarly, older women aged 35–49 years were 7.53 (95% CI = 5.11–11.08) times more likely to have had a CS birth than younger women (15–24 years). Parity had a direct relationship with CS delivery. Women who had two births and at least 3 births were 0.53 (95%CI = 0.38–0.73) and 0.31 (95%CI = 0.22–0.43) times, respectively less likely to have a CS delivery than women with one birth.

Regarding education, secondary educated and higher educated mothers were about 1.65 (95%CI = 1.15–2.36) and 2.17 (95%CI = 1.26–3.74) times, respectively, more likely to have a CS birth relative to uneducated women. CS delivery among women with primary education were not significantly different from uneducated women (AOR = 1.59, 95% CI = 0.98–2.59).

Concerning wealth quintile of women, the odds of having a CS delivery was 2.76 (95%CI = 1.77–4.28) times and 4.38 (95%CI = 2.83–6.77) times higher among richer and richest women respectively, than the poorest women. Likewise, middle-class mothers were 2.13 (95%CI = 1.43–3.18) times more likely to have CS delivery relative to the poorest mothers. No statistically significant difference in the likelihood of CS delivery was found between poorer and poorest women (AOR = 1.36, 95%CI = 0.89–2.06).

The study findings point to wealth-related inequity in CS birth. The analysis revealed higher CS birth among rich mothers when compared to the poor (rich: poor ratio = 5.5). As shown in Fig. 1, richer and richest women had a higher CS rate than average national CS rate. A positive concentration index value of 0.172 was calculated, which indicates more concentration of CS delivery among wealthier women (Fig. 3).

**Fig. 2:**
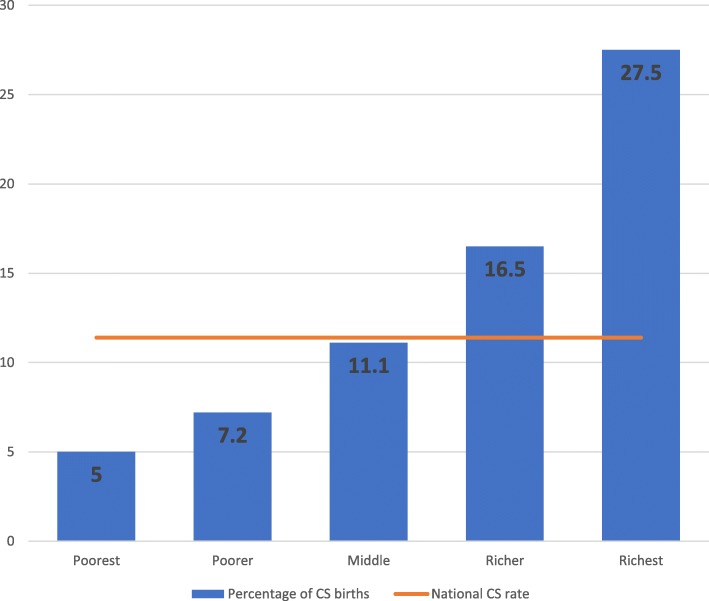
Distribution of caesarean delivery by wealth quintile, GDHS data 2014

Likewise, the concentration curve displays a curve below the equality line representing greater frequency of CS births among the affluent group (Fig. 2). Finally, based on Koolman and Van Doorslaer’s methodology, 12.9% of CS deliveries should be shifted away from the richer group to address the unmet CS delivery needs among less privileged women to achieve a concentration index of zero. The use of concentration curve and index is only to illustrate the extent of income-based inequalities in utilization. However, more research is needed to determine how inequitable this utilization pattern is. The goal is not necessarily to reach a concentration index of zero. If CS acted as a luxury good in an economic sense, then it would be expected that richer women would use more of it. More affluent women having higher rates of CS is not necessarily a problem in terms of equity if these CS surgeries are not paid for using public money and if poorer women have access to needed CS deliveries.

**Fig. 3:**
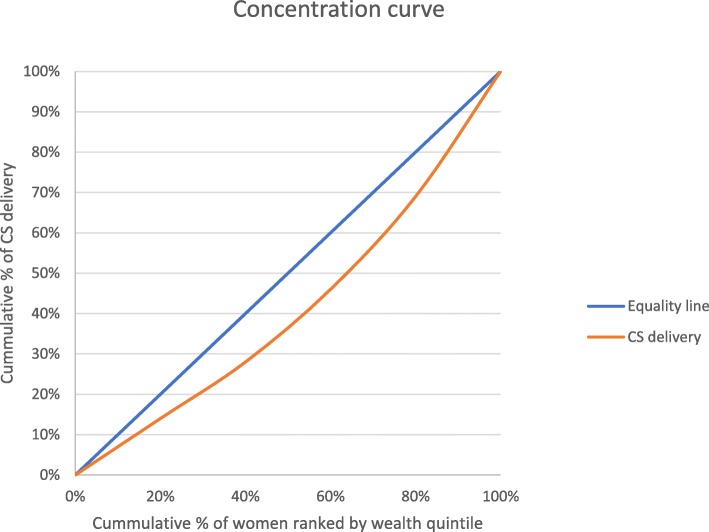
Concentration curve for caesarean delivery, GDHS data 2014

## Discussion

This study contributes to the literature that examines inequalities in the use of obstetric care in low and middle-income countries. Specifically, the study sought to examine the socioeconomic inequities in CS delivery among Ghanaian women of childbearing age. In this study, the total CS rate of 11. 4% was higher than 6.59% reported in a similar study in Ghana [30] and within the optimal levels of 5–15% [6, 15]. In addition, the CS rate from this study was higher relative to the 7.3 and 3% CS rate estimated for Africa and West Africa respectively [14].

Results of the multivariable data analyses of 4294 women identified strong associations between CS birth and some predictors including mother’s age, parity, education level, and wealth quintile.. In this current study, marital status of the women was not significantly associated with CS birth, this result is consistent with a similar study in Ghana [30]. Also, religion was not a significant predictor for use of CS deliveries in this study; this is in contrast with the results of a similar study from a different geographical context that found a strong association [44]. Similar to the findings of this research, a study in Ethiopia reported that ethnicity of women was not significantly related to CS delivery [22]. A study conducted in China [45] and Egypt [25] posited that urban dwellers were more likely use CS delivery than their rural counterparts, but this research found a significant relationship between place of residence and CS birth at univariable stage, and the effect was attenuated in the adjusted model. The decentralization of the Ghanaian healthcare and effective referral system may play a role in the finding.

### Age

The relationship between CS birth and age has been studied extensively in the literature with considerable mixed findings. A study conducted in Egypt found that younger (30 years and below) women were more likely to have a CS birth [25] whilst other authors observed higher likelihood for CS delivery among older women [24, 26, 46–48]. The latter finding is consistent with the results of this study. This result could be explained by natural physiological and anatomical changes accompanying aging which expose older mothers to an elevated risk of pregnancy and delivery related complications [49–51]. These physical changes coupled with a higher rate of request for CS delivery by these mothers [49] could be the main reasons for the higher rate. Also, the older women may perceive caesarean section as a safe delivery option to protect their fetus after a long period of conception difficulty, and the fear of delivery pains and losing of baby. Further studies into the level of maternal requests for CS delivery among older women are needed to better understand the medically unnecessary CS births in the cohort. Alternatively, perhaps uneasiness and fear associated with CS delivery could deter particularly younger mothers as reported in similar studies in Nigeria [52] and Tanzania [53]. Also, young mothers may refuse CS delivery because of the risk of repeated CS births and the complications associated with more than 3 repeated CS as reported in a study [54]. Moreover, the complications and repeated CS may restrict the number of children born by these young women because health professionals do not recommend childbirth after 3 successive CS.

### Parity

The association between woman’s parity and the likelihood to have a CS delivery has been long-established in body literature. Conventionally, mothers with higher birth order are less likely to have a CS [24, 25, 33]. The results of the current study are consistent with previous research in this area. First time mothers have a higher likelihood of having a CS delivery [26] probably due to fear of labor pain [28]. On the other hand, mothers who had given birth before have a lower likelihood of having a CS delivery until the fifth birth [55]. This lower CS delivery among multiparous women could be explained by their previous delivery type as well as level of satisfaction of the obstetric care received. Supporting this possible explanation, a study in Burkina Faso [56] reported that mothers with a normal delivery experience might reject CS delivery even when it is medically recommended because of the guilt of not delivering naturally and the risk of possible future caesarean sections. Also, other studies have found that women with previous caesarean delivery have a higher likelihood of repeated CS [32, 57].

### Education

Earlier studies which examined the association between maternal education level and CS delivery have had mixed findings. Though a study found no statistically significant association between the likelihood of CS delivery and education level of the mother [25] other studies have also revealed that education level of mothers was strongly linked to the likelihood of CS delivery. Moreover, a study conducted with a smaller sample size at northern Ghana reported lower risk of CS delivery among women with secondary or higher education [58]. Conversely, this study’s findings are consistent with previous studies in Bangladesh [24], Brazil [48], Thailand [27], Pakistan [26] and China [59] which found that highly educated (secondary and postsecondary) women were more likely to have a CS delivery than women with no formal education. This finding could be explained by the enhanced ability of women with secondary and postsecondary to better access obstetric care due to their autonomy and ability to take decisions about their health [60] and understanding the importance of CS interventions if needed. On the other hand, women with no or limited education may have limited knowledge and/or misconceptions about CS which could discourage use, and even may not report to health facilities amidst delivery complication for the CS procedure [61].

### Wealth quintile

Research into the relationship between wealth quintile and CS delivery has been well established in literature. With the exception of the finding by Robelo et al. [46] that an inverse association exists between improved wealth quintile and CS delivery rate, most other authors have reported that the likelihood of CS delivery increases with better wealth quintile [11, 26, 45, 59, 62–64]. The results of this study are consistent with the overall literature in general and with similar studies such as the study from Mozambique that reported underuse of CS delivery in less affluent mothers [65] and another study from Bangladesh that revealed about 2.5 times likelihood of CS delivery among wealthier women relative to the poorest [24]. This study’s finding of higher likelihood of CS delivery among richest, richer and middle-class women could be the result of the costs associated with CS birth. Though CS delivery is covered in Ghana’s free maternal health services policy [66, 67] Ghanaian women may still incur indirect costs including transportation [68], and unapproved fees from health professionals as well as service expenses outside the policy [69]; These CS related costs could play an important role in preventing poor women from accessing CS care at health facilities. Type of health facility accessed for obstetric care has been reported to be associated with use of CS for delivery in international studies, showing a higher likelihood of CS in private facilities [32, 70], and a higher likelihood of CS deliveries to be medically justified in public hospitals [71]. However, this study did not include place of delivery as a explanatory variable.

Further, affluent women may tend to have a higher likelihood of requesting CS delivery [4] because of perceived lower risk [72]. Contrary to the findings of a study by Hou et al. [59], this research found no significant difference in the likelihood of CS delivery between women in the two lowest wealth quintiles.

Finally, on wealth-related inequities in CS delivery, the rich versus poor ratio of 5.5 computed in this study clearly demonstrates a pro-rich CS intervention uptake, though lower than the ratio of 7.52 and 7.73 reported in Bangladesh [44] and Namibia [41] respectively. Further, the concentration curve and the concentration index reported in this study testify to the degree of inequality in the use of CS services as found in similar studies [41, 44].

### Strengths and limitations of the study

This study used a large nationally representative population-based data to investigate the factors associated with inequalities in the utilization of CS delivery among women in Ghana. However, a few limitations of the study data were identified. First, recall bias could arise because the survey relied on self-reporting. However, the question regarding CS birth was restricted to recent birth within 5 years preceding the survey to limit recall bias. Also, the GDHS did not include data on medical need for CS and hence further population-based studies are required to investigate association between CS delivery and medical need factors. Finally, as a result of lack of data on type of CS and place of birth, the study could not distinguish between elective and emergency CS and whether CS delivery occurred at a public or private facility.

## Conclusions

Even though Ghana has achieved a CS rate of 11.4%, which is consistent with WHO recommendations, it still has a high maternal mortality rate; this study’s finding of large inequalities in the use of CS based on wealth and education helps provide an explanation for this apparent contradiction. The importance of generating evidence for the presensce of socioeconomic inequalities in the use of CS in Ghana is that it directs public policy to go beyond aggregate level indicators and critically examine caesarean delivery distribution. Moreover, despite Ghana’s free maternal healthcare interventions, poorer women had much lower use of CS holding everything else constant which indicates that removing fees alone may not be sufficient to adequately improve access to CS for poor women. This policy implication is similar to that proposed by other researchers who have proposed paying utmost attention to socially disadvantaged women to minimize inequalities in the use of CS delivery. On the other hand, while, the underuse of CS among poorer and lower educated women raises serious concerns about access to a life-saving surgery, the possible medically unjustified overuse of CS delivery as income and education increases raises a different set of concerns, which require targetted health policy interventions to achieve more appropriate use of CS among richer and more educted women.

## Data Availability

The datasets analyzed during the current study are available in the MEASURE DHS repository, https://www.dhsprogram.com/data/dataset_admin/login_main.cfm. ^[GHIR72DT]^

## Abbreviations

AIC: Akaike’s Information Criterion
ANC: Antenatal Care
AOR: Adjusted Odds Ratio
AUC: Area under the curve
CI: Confidence Interval
CS: Caesarean section
GDHS: Ghana Demographic and Health Survey
GSS: Ghana Statistical Service
OR: Odds Ratio
PHC: Population and housing Census
ROC: Receiver operating characteristics
SD: Standard deviation
VIF: Variance Inflation Factor
WHO: World Health Organization
